# The consequences of being labelled ‘looked‐after’: Exploring the educational experiences of looked‐after children and young people in Wales

**DOI:** 10.1002/berj.3283

**Published:** 2017-04-26

**Authors:** Dawn Mannay, Rhiannon Evans, Eleanor Staples, Sophie Hallett, Louise Roberts, Alyson Rees, Darren Andrews

**Affiliations:** ^1^ Cardiff University UK

**Keywords:** education, foster care, looked‐after children and young people, unintended consequences

## Abstract

The educational experiences and attainment of looked‐after children and young people (LACYP) remains an issue of widespread international concern. Within the UK, children and young people in care achieve poorer educational outcomes compared to individuals not in care. Despite proliferation of research documenting the reasons for educational disadvantage amongst this population, there remains limited empirical consideration of the lived experiences of the educational system, as perceived by LACYP themselves. This paper draws upon qualitative research with 67 care‐experienced children and young people in Wales. The sample was aged 6–27 years, and comprised 27 females and 40 males. Participants had experienced a range of care placements. Findings focus on how educational policies and practices alienate LACYP from dominant discourses of educational achievement through assignment of the ‘supported’ subject position, where children and young people are permitted and even encouraged not to succeed academically due to their complex and disrupted home circumstances. However, such diminished expectations are rejected by LACYP, who want to be pushed and challenged in the realisation of their potential. The paper argues that more differentiated understandings of LACYP's aspirations and capabilities need to be embedded into everyday practices, to ensure that effective educational support systems are developed.

## Background

The number of looked‐after children and young people (LACYP) in Wales stands at 5415, with this figure having increased by 20% in the past 10 years (Welsh Government, 2015a). The educational experiences and attainment of LACYP remains a concern, as they are reported to perform less well than the general population across a range of outcomes (Jackson, 2010; O'Higgins *et al*., 2015; Sebba *et al*., 2015). International data indicates that completion rates for primary and secondary education are lower in comparison to the general population (Vinnerljung & Hjern, 2011), with levels of academic achievement being systematically poorer (Courtney & Dworsky, 2006; Berger *et al*., 2015).

National data for Wales reports that in 2015, 18% of LACYP achieved the Key Stage 4 threshold (five GCSEs Grade A*–C including English or Welsh first language and mathematics), compared to 58% of the total student population (Welsh Government, 2016a). This attainment gap widens as LACYP progress through key educational stages and then transition into higher education (Stein, 2012). Such disadvantage has serious consequences for future life chances (Jackson, 1994), and although educational attainment is not the only predictor of success (Berridge, 2012), the increasingly competitive employment economy emphasises the importance of qualifications and skills (Brown *et al*., 2013).

There has been a proliferation of legislative action in response to the educational outcomes for LACYP, both within Wales and the UK (see The Children Act 1989, The Children Act 2004, The Children and Young Persons Act 2008, The Social Services and Well‐being (Wales) Act 2014). Since devolution, in 1999, the Welsh Government has progressed a plethora of targeted educational approaches, with existing provisions being summarised in the *Raising the ambitions and educational attainment of children who are looked after in Wales strategy* (Welsh Government, 2016b). These include: establishment of dedicated local educational coordinators to monitor progress; LACYP education support workers to provide catch‐up support; designated teachers in school to support LACYP; and the Personal Education Plan, which is soon to be reconfigured as the Individual Development Plan (Welsh Assembly Government, 2007; Welsh Government, 2016b). Financial support offered to local authorities has also been provided in various forms, such as the RAISE (Raising Achievement and Individual Standards in Education) programme and, more recently, the Welsh Government's Pupil Deprivation Grant (Welsh Government, 2015b).

Despite these government initiatives, educational outcomes for LACYP have yet to improve significantly, and at times the attainment gap appears somewhat intractable. This raises the fundamental question of whether current policies respond fully to the complex causes of the problem (Brodie, 2010; Berridge, 2012; Stein, 2012). Multifarious explanations for educational disadvantage have been proffered, amidst suggestions that features of the care system may be central to explaining the poorer outcomes of this population. These include: inadequate information transfer between agencies, which can restrict enrolment in schooling (Zetlin *et al*., 2004); frequent movement of home and school placements, which disrupts learning opportunities (Ferguson, 2007; Jackson & Cameron, 2010; Pecora, 2012); and a perceived lack of prioritisation of education for LACYP, which is compounded by inadequate accountability or monitoring (Zetlin *et al*., 2004; Ferguson & Wolkow, 2012)—all of which can impact subjectively (Hallett, 2015).

A range of non‐random factors, including deprivation, family breakdown, special educational need status and childhood trauma, which predict entry into care but which are extrinsic to the care experience, may also independently explain educational disadvantage (Berridge *et al*., 2008; Welbourne & Leeson, 2012; Sebba *et al*., 2015). Yet, regardless of advancement in the theorisation and empirical substantiation of the reasons for LACYP's poorer educational outcomes, it remains apparent that these explanations often omit the lived experiences of young people themselves.

This paper explores the educational experiences of LACYP, paying particular attention to how the assignment of the ‘looked‐after’ subject position, through enactment of school policies and practices, can confer unintended consequences and exacerbate educational disadvantage. These experiential accounts were facilitated by creative activities, which allowed participants to reflect in detail on the micro interactions of their schooled lives. This allowed space for thinking through their subjective, mundane, but important, experiences that operate alongside, and interact with, more structural changes. Focusing on the commonplace, ordinary and routine aspects of school life centralised the ways in which subject positions are made and remade, and their educational impacts. The following section sets out the theoretical framework that was adopted to explore these subject positions, their ascription and their rejection.

## Theorising the attainment gap: Assignment of the ‘failing’ subject position

Educational institutions position students in relation to the dominant discourse that prescribes their construction of the desired and desirous student (Hall *et al*., 2004). This discourse may be structured by notions of a holistic and pastorally driven educational experience (Evans, 2015), but predominantly encompasses the privileging of academic attainment, in alignment with the commodification and standardisation of achievement in the form of league tables and ascribed grades (Benjamin *et al*., 2003; Hall *et al*., 2004; Donnelly, 2015; Evans, 2015). Hierarchical binaries emerge in response to such discourses, with indices of difference inevitably being inscribed. As Benjamin (2002) maintains, for the successful subject position to flourish, the failing subject is necessarily brought into being, as both are relative constructs. Assignment of the non‐academic subject position has the potential to be detrimental, as—in alignment with Merton's (1948) labelling theory, which was latter explored in Rosenthal's and Jacobson's (1968) seminal study of teachers’ expectations and the subsequent consequences for students—this can lead to a ‘self‐fulfilling prophecy’.

Empirical substantiations of such phenomena can be found in Benjamin's (2002) ethnography of young women deemed to have special educational needs (SEN), which depicts how educational practices reify differences between students whilst disenfranchising those with additional learning needs from dominant discourses of academic success. Further ethnographic work by Ivinson and Murphy (2007) documents how pedagogic practices institute regimes of gendered learning that alienate females. Indeed, the authors suggest that the gaze of teachers is imbued with the cultural legacies of science subjects, which are characterised by a male rationality, and the resulting gendered territorialisation of knowledge denies females legitimacy in learning and inhibits their academic progress.

Concurrently, concern around how intervention responses address the prospects of the ‘failing’ subject position may actually compound the problem, thus serving as a ‘cure that harms’ (Dishion *et al*., 1999; McCord, 2003; Wiggins *et al*., 2009). Research conducted by Evans (2015) explored how labelling of students in need of participation in a social and emotional learning programme was often interpreted negatively, with students perceiving intervention as a rejection by teachers, which led to an exacerbation of anti‐school behaviours and further academic disengagement.

Accordingly, children and young people who are routinely and clearly ascribed the label of ‘in care’ by the social and educational system remain at risk of being assigned the ‘failing’ subject position. There has been extensive documentation of LACYP encountering unsupportive professional or carer practices that further inscribe indices of difference by stigmatising their care status and undermining their expectations for achievement (Harker *et al*., 2003; McLeod, 2010). Equally, as Berridge (2012) has emphasised, the behavioural difficulties and complex learning challenges experienced by LACYP have been inadequately addressed, with many of the individuals assigned statements of SEN being misunderstood, which has routinely led to the insufficient provision of appropriate support and exacerbation of educational problems (Fletcher‐Campbell & Archer, 2003). This paper aims to explore LACYP's contemporary educational experiences and how being labelled as ‘in care’ impacts upon their positionality within dominant discourses of academic success, and the implications that this label has for their attainment.

## Methodology

The data presented in this paper were generated as part of a Welsh Government‐commissioned study to explore the educational experiences, attainment and aspirations of LACYP in Wales (Mannay *et al*., 2015). The study was conducted through the collaboration of Cardiff University, The Fostering Network,^1^ Voices From Care Cymru^2^ and Spice Innovate.^3^

67 LACYP in Wales participated in the study: 22 in primary school (aged 6–11); 17 in secondary school (aged 11–16); 26 who had completed compulsory education with mixed engagement with further education (aged 16–27); and 2 in higher education. Of the participants, 27 (40%) were female and 40 (60%) were male. This sample did not directly reflect the gender balance of the public care population. As at 31 March 2015 there were 5615 children in public care in Wales: 2595 girls (46.3%) and 3020 boys (53.7%) (Welsh Government, 2015a).

All participants had attended mainstream schools and experienced a range of care placements: foster care (*n *=* *52); foster, residential and kinship care (*n *=* *4); foster and residential care (*n *=* *7); foster and kinship care (*n *=* *1); foster care and semi‐independent (*n *=* *1); residential care only (*n *=* *1); and unspecified (*n *=* *1). The number of care placements ranged from 1 to 24. The mean average of placements for primary school children was 1.95, for secondary school children 2.92 and for the aged 16–27 group, 10.83.^4^ However, 12 LACYP did not record how many times they had moved placements.^5^

Participants were purposively recruited through the networks of the research partners and other external agencies. For primary and secondary school‐aged participants, foster carers were invited by The Fostering Network, through a mail out to their members, to bring their foster children to attend an activities event day where research would also be undertaken. Of the participants who were post‐compulsory education, 17 were recruited through Voices From Care Cymru, where they served as volunteers or regularly attended events. A further 7 were recruited through local authority groups for LACYP or care leavers. The 2 participants in higher education were recruited via an email circulated by the Care Leavers Activities and Student Support (CLASS) Cymru Network and by emails to individual key contacts for care leavers at Welsh universities.

Research with primary and secondary school‐aged LACYP were conducted during four separate event days organised with assistance from The Fostering Network. Three events were hosted in South Wales and one in North Wales. Research involved the conduct of one‐to‐one interviews with integrated creative methods. This reflects the mainstreaming of a commitment to children and young people's participation in research about them, whilst taking advantage of the increasing variety of techniques used to foster that participation (Lomax *et al*., 2011; Mannay, 2013, 2016; Kim, 2015). Use of such approaches was intended to avoid the recreation of social work encounters in which young people's accounts may inform fundamental and difficult decisions about their lives, including removal from their birth families, thus potentially instilling a resistance to share subjective views and narratives. In contrast, providing participants with the power to lead the research activity through the creation and discussion of visual artefacts creates a more neutral space where they might engage with the research on their own terms.

The visual and creative methods employed included sandboxing and emotion sticker activities. The sandboxing activity was designed to elicit participants’ ideas about their aspirations and participants created scenes in sand trays with figures representing their future and what they wanted to do, be or achieve. The drawing and emotion sticker activities provided an opportunity for participants to reflect on their experiences of education. The visual activities were followed by individual elicitation interviews with a member of the research team, where children described what they had made. This was supplemented by an interview schedule about educational experiences and aspirations, which was used to discuss any areas that were not covered in the conversations around the visual activities.

Research with post‐compulsory education participants involved focus groups, which were conducted in South and North Wales. Six focus groups were undertaken. Focus groups were conducted by care‐experienced peer researchers with the support of the research team. Peer researchers are increasingly encouraged in the conduct of research with often marginalised groups of young people due to the potential to avoid the power imbalance that can often exist between adult researcher and young participant (Stein & Verweijen‐Slamnescu, 2012; Lushey & Monroe, 2014). We also felt that a care‐experienced researcher would be more engaging and relatable, whilst their ‘insider knowledge’ enhanced the research process by sensitising focus group questions to the needs of the participants. Following conduct of the focus groups, it was apparent that none of the participants were in higher education or were considering higher education. As it was important to include these experiences, two semi‐structured telephone interviews were undertaken with care‐experienced participants in higher education; these interviews were led by a member of the research team.

Interview and focus group data were transcribed verbatim and analysed concurrently throughout data production, allowing codes, categories and themes to emerge from the empirical data produced with LACYP. Data were analysed using an inductive and deductive approach, creating overarching thematic categories and analytical themes arising from coding and categories across the data sets. The visual materials, which were photographed at the point of data production, acted as tools of elicitation, rather than objects of analysis per se. However, they were considered in the analysis to clarify and extend the associated interview transcripts.

Initial codes were formed, and related codes grouped or merged from across each data set to create a coding framework of coding themes and sub‐themes. For example, ‘care‐related appointments in school’, ‘called out of lessons’, ‘arriving in taxi’, ‘parents’ evening’ formed part of the theme ‘visible difference’. Analysis was undertaken by three members of the research team, and was accompanied by an iterative process of reviewing and cross‐checking these emerging themes and interpretations with relevant literature, concepts and theory, to allow for the incremental development and testing of analytical concepts. Ethical approval for this study was provided by Cardiff University.

## Findings

The first part of this section explores how the label ‘looked‐after’ is inculcated in care‐experienced individuals as they transition from childhood into youth and early adulthood, amidst an emerging awareness of their potential positionality outside dominant discourses of success. The second part continues by considering the policies and practices enacted by schools in order to create alternative subject positions for LACYP that are divorced from academic attainment and progression. The third part examines how young people actively resist their ‘looked‐after’ label and the ascribed trajectory of failure, demonstrating an acute determination to realise their potential and achieve academic success. There is also a focus on the internal and external resources that make this agentic subject position possible.

### From similarity to difference: Inscription of the ‘looked‐after’ label

Narratives of participants’ educational experiences were imbued with feelings of being either the same or different from those who have not experienced care. Children in the study did not delineate themselves as being different, and the label of ‘looked‐after’ did not form a central aspect of their identity. They voiced aspirations for their future with enthusiasm and confidence, expressing career ambitions similar to those desired by non‐LACYP, including the professional roles of vets, doctors, teachers and architects (Davey, 2006; DCSF, 2010).

I think be a doctor and have a car. (Jessica,^6^ aged 9)

I want to be an architect … because I like art and most of my family are builders. (Hulk, aged 12)

I want to go to college. Once I've finished college I'll go to university to learn about geography. (Roxy, aged 12)

I want to be a teacher. When I've finished university, I'm going to find a school and ask the headmistress if I can join. (Imogen, aged 11)

However, despite a lack of overt acknowledgement of their identity of being in care, some children hinted at the importance of education and career for creating and maintaining a family, with emphasis on keeping everyone together:

I wouldn't mind making a lot of money, just in case I have a family so we're actually able to look after them and to keep them safe. (Bishop, aged 11)

Although these younger participants did not explicitly position themselves in contrast to their non‐looked‐after peers, discussions of future aspirations for stability and safety may reveal an underlying concern regarding their disrupted home circumstances or possibility of future placement moves. We are unable to make comparisons with non‐looked‐after populations. However, removal from the family home and further placement moves are central to the looked‐after experience; and this potential concern is worth noting in relation to both its emotional impacts and the reported links between disrupted home circumstances and educational attainment (Pecora, 2012; The Fostering Network, 2014).

In juxtaposition to the primary school‐aged children, young people displayed an acute awareness of their status as being ‘looked‐after’ and how this label invariably demarcated them as being different by both professionals and peers. Through the introduction of this difference a hierarchical schema of identities inevitably took hold, with the LACYP subject position being imbued with negative connotations that were often synonymous with the notions of ‘troubled’, ‘scroungers’ and ‘of concern’. Even where participants expressed hope and optimism for their future, they remained aware of the identity that society had inscribed for them, and were continually struggling with the assumption that they were failures and problems in the making. The majority of young people expressed frustration at being viewed and understood through the lens of being ‘looked‐after’ (see also Hallett, 2015). Thus, they were keen to reject this notion of difference, which was grounded in the restrictive and homogenised marker of LACYP, whilst simultaneously being invested in defining themselves as unique and complex characters:

We don't want people to be ‘looked‐after’, you want to be a normal kid too you know because it's only one, its only label of you. (Female participant, focus group^7^ )

I hate people feeling pity for me. I'm just a normal child, like … I'm in foster care, it doesn't mean you're just like some pity child. (Male participant, focus group)

Inscription of such indices of difference also manifested within the school context, with the label ‘looked‐after’ assuming a prominent role in their educational experiences. Young people described incidents of attending local authority care (LAC) reviews and meetings with social workers conducted at school, in rooms where they were visible to passing peers. On occasion, social workers would call them out of class to attend meetings, or support workers would sit with them during lessons. These events were seen as exposing their personal lives, whilst making their differences from other students visible:

I don't know bad bit was like the LAC reviews and whatever because the teachers kind of knew that you were in care and whatever and that, they all were, people would be like, ‘oh why are you going with Miss So‐and‐so? (Nadine, aged 21)

I just didn't want it, I was like I don't need that, it's singling me out and its making me seem special when I'm not, I'm a normal person. (Female participant, focus group)

Any meetings, if they are necessary, should be held outside of school time, not just at a time that is convenient for the professionals. (Female participant, focus group)

Meetings in school time were not only detrimental in terms of being seen as different, they also impacted on LACYP's emotional health and the routines of the school day. Many of the participants missed out on education because of these meetings and reviews, which made them fall behind with work and disrupted their school days. Being removed from lessons also created stress and anxiety, as meetings were often emotive and returning to the class meant facing questions from peers about the nature of the absence. Consequently, a meeting of 45 minutes might lead to disruptions in the days leading up to the review and those following the meeting. Hence, through these routine practices and performances, the differences attributed to LACYP become reified and even amplified.

### Outside dominant discourses of success: The ‘supported’ subject position

Whilst young people became increasingly aware of their construction of being different, they also considered how such entrenched notions of difference led to their positioning outside dominant discourses of success within schools. Such sentiments were not evident amongst the primary school‐aged children, whose assessment of school was descriptive and evaluative. They spoke of friends and school staff, with each identifying teachers who were nice to class, and those who were mean to everyone. Some students spoke of school as an enjoyable experience, such as Caitlin (aged 10) who claimed it was ‘*great, super, supercalifragilisticexpialidocious*’. Meanwhile Musa (aged 8) maintained that it was ‘*Work, work and work. School is a bit boring*’.

In contrast, young people reflected at length on their educational experiences, and how this was informed by their positioning outside discourses of academic attainment due to their looked‐after status. Some participants did provide best‐practice case examples, where teachers had supported and encouraged their aspirations, but most documented professionals’ low expectations for their achievement and career trajectories (Jackson & Sachdev, 2001; Fletcher‐Campbell & Archer, 2003; Berridge, 2012):

Various foster carers and people to do with the care system were like ‘oh people in care don't go to into higher education’. I wish social services would focus less on that because a lot of them have social work degrees so who are they to be telling anyone else that they're not worthy of university? It's like they don't believe that children in care will do anything. And so if they don't believe it, then how is anyone going to believe it about themselves? (Female participant, focus group)

I remember telling the head of sixth form that I wanted to be a teacher and whatever, and she said you should look at college courses and stuff, and I was just like no I want to go to university. (Female participant, focus group)

Some teachers were like openly against us, you know, they were like ‘oh there's no point in trying with them’ sort of thing. (Female participant, focus group)

Participants perceived these expectations to be grounded in professionals’ assumptions that being looked after was linked to lower intellectual capabilities, combined with an awareness of the intimate and complex aspects of their home life. Young people felt that the dominant response to such knowledge and assumptions was pity and (sometimes false) sympathy. This informed their exceptional treatment, where they were routinely afforded numerous allowances, negating them being academically challenged, due to already being exposed to such complex and difficult life circumstances:

As soon as I went into care, then went back to school and my teachers majority of them treated me completely different, because I was in care they moved me down sets, they put me in special help, they gave me – put me in support groups. And I was just like I don't need all this shit, I've only moved house, that's it I was like yeah I might be in care but the only difference to me is I've moved house, that's it … they looked at all my papers and where I was in my levels and that and they was like you're more than capable of being in top set but we don't think you're going to be able to cope. (Female participant, focus group)

If we was a child that wasn't in care we'd be made to sit there and get on with our work or something, like if we wasn't having family problems if we were just in a mood. Then some children that are in care could go into school and just go, ‘I ain't doing this today’, and then they'd just be left to the side because they think it's just family problems, but it might not be, it might just be them being a normal child. (Female participant, focus group)

Such concessions can arguably be interpreted as an effort by schools to be responsive to the needs of students. However, responding to the label of ‘looked‐after’ through ascription of the ‘supported’ subject position potentially confers unintended harms by restricting opportunities for academic achievement.

Solutions for schools’ policies and practice were proffered. Participants acknowledged that they required additional support on occasion, and described the importance of being listened to or having someone understand their sometimes resistant or disruptive behaviours. However, they predominantly felt that the most constructive approach was for schools to draw LACYP into the prevailing discourses of academic success by encouraging them to participate in lessons or schooling, and push them academically:

It's about motivation. All you need is a good kick up the arse. And I think if somebody had given that to me when I was 16 or 17, I would probably have been like ‘right, that's it I want to, I'm going to do something with my life. (Male participant, focus group)

Such remedies are not only instructive, but importantly illustrate some of the tensions and nuances associated with schools supporting LACYP and offering an ‘easy option’ or ‘special help’. Whilst many thought it important that schools offer additional support, they felt it should be developed in consultation with the individual, so that presumptions about their needs and experiences are not made. Participants also indicated the need to offer universally available resources, such as a designated person or safe room, to all students in order to avoid the label of ‘looked‐after’ being interpreted as an indicator that an individual is of concern or problematic. Such sentiments resonate with the broader literature pertaining to the unintended harms of targeted interventions, where negative labels are assigned to participants (Evans *et al*., 2015), alongside those that emphasise the need to involve young people in decisions about their care (Sennett, 2003; Hallett, 2015).

### Reclaiming success: Resisting the ‘failing’ subject position

Amidst participants’ acute awareness of how their assignment of the ‘supported’ subject position restricted their opportunities for academic attainment, they also demonstrated how they challenged and resisted the label ascribed to them by teachers and other professionals. Nadine and Megan were the only participants with experience of university. Therefore, it is important to reflect in depth on their extended educational trajectories and the barriers they overcame to access forms of higher education.

My sixth form leader, she basically told me that I had no chance of getting into university … she made me feel quite rubbish sometimes … and I was just like no I want to go to university. So it was kind of like I don't know, like that will show her that I could get there. (Nadine)

Despite Nadine's apparent determination and resilience to the responses of others, her positioning outside academic success was emotionally difficult and could undermine her belief in her own educational abilities.

When I'd come home crying because my teacher said I'm not going to be able to do it (my foster carer) used to say no you can, you can, she was really supportive … I was part of the Looked‐After Care Council and we went to a conference thing and they were saying about students in care like not achieving what they should and whatever, and saying that only 1% like go to university and whatever. And my foster carer … she was like, ‘you're going to be that 1%’. And I don't know it kind of just put a little bit of more belief in me and it just made me want to do it that little bit more. (Nadine)

To resist the positioning of educational failure, individuals required the support and belief of other salient adults in their lives. Accordingly, Nadine centralises the importance of her own agency, her relationship with her foster carer and her involvement with the Looked‐After Care Council, which combined to enable a rejection of the educational stigma associated with being ‘looked‐after’. Despite evidence of young people's capacity to circumvent the subject position of academic failure, it is important to consider the social and cultural capital afforded to Nadine, whilst acknowledging that not all LACYP have the same foundational base of support, experience or knowledge:

Without my foster carer I wouldn't be where I am today … her children went to university as well so she was, she was all for it whereas I know other foster carers maybe who had not had the same experiences as my foster carer so it is important definitely. (Nadine)

Therefore, although LACYP can actively resist academic failure, it is more difficult to successfully negotiate the educational terrain without these networks of support, as illustrated by Megan's account:

I'd always wanted to go. Just when college and school messed up like the first time, I kind of just thought that I'd wait until I was a mature student and figure out what I actually wanted to do. Like mainly because everyone always told me that I couldn't. So it was just a kind of thing of I wanted to go just because I could. (Megan)

Like Nadine, Megan also resists the low expectations of ‘*everybody*’, replacing the attribution of ‘*couldn't*’ with the binary opposite of ‘*I could*’. However, without a supportive framework, Megan's early educational account is one of conflict, educational failure and a representation of dominant self‐fulfilling prophecies for LACYP. Again, this centralises the powerful influence of expectations and reinforces the argument raised earlier that when the label of ‘looked‐after’ is interpreted as an indicator that an individual is educationally problematic, this creates barriers for their progression. Nevertheless, drawing on her own agency, Megan actively sought out an institution that communicates a commitment to, and belief in, care leavers in their online promotional materials.

That was one of the main reasons that I applied to [this] university is because they're one of the only universities that mentions anything about care leavers on their website. Like they've got a whole video about it and yeah I just kind of like emailed [support staff] before I came and she was just kind of really friendly and helpful and was just basically like if you ever need anything, just stop by. I emailed her as soon as I knew that I was coming here … she supported me the whole way through these two years. (Megan)

Enrolled at a university where these assurances of support are actualised with encouragement, help and assistance, Megan was able to complete the first two years of university. Consequently, despite earlier negative experiences of education, Megan did not passively accept alignment with the failing subject, which was generated by the key actors responsible for her care, but sought out alternative pathways to higher education. However, whilst Megan demonstrates clear successes, she equally acknowledges the invisibility of much support and resources within higher education, which can inhibit LACYP transgression of the failing label:

They need to like advertise it more, the support that is actually there, particularly the financial which they keep very well hidden. (Megan)

Taken together these accounts evidence the agency of LACYP to challenge their positioning as ‘failing’ subjects, drawing on their ‘looked‐after’ experience and belief in their own abilities. However, higher education remains a difficult terrain to navigate, not least because of the expectations communicated to Nadine and Megan that university is not for ‘people like them’. In the cases of Nadine and Megan, access has been facilitated through the determined exercise of their own agency, combined with the support of an adult whose conceptualisation of care leavers is premised on the educationally successful subject. Yet, whilst the achievements of these individuals may be commended, it remains clear that not all LACYP will have access to such support systems.

## Discussion

This paper explores children and young people's lived experiences of education, in relation to their ‘looked‐after’ status, considering their understandings of educational disadvantage in addition to potential remedies. The problematic nature of labelling and the unintended educational consequences of support systems that aim to ‘look after’ children and young people in care were key features in their accounts and recommendations. In resonance with studies documenting hierarchical binaries within educational institutions (Benjamin *et al*., 2003; Ivinson & Murphy, 2007; Evans, 2015), where the relational subject positions of ‘successful’ and ‘failing’ are routinely assigned to students, LACYP are often positioned outside dominant discourses of success. However, inculcation with the ‘failing’ subject position is a nuanced process, often couched in an expression of concern and sympathy by teachers and broader institutional structures. Indeed, it may be more accurately defined as the ‘supported’ subject position. Within this process, LACYP are already considered to have challenging and often chaotic life circumstances, and are excluded from encouragement to strive academically in order to mitigate against the risk of further stressful life events.

Discussions pertaining to the ‘supported’ subject bring sharply into focus concerns around the unintended harms of targeted intervention with vulnerable or at‐risk individuals (Dishion *et al*., 1999; McCord, 2003; Wiggins *et al*., 2009; Evans, 2015). The additional resources and exceptional treatment provided to LACYP were often considered to be stigmatising in their foregrounding of students’ differences from the rest of the school population, whilst occasionally diminishing young people's future expectations for themselves.

The accounts of younger children were aspirational and, despite some references to the problematic nature of moving home and school changes, they documented their educational journeys as a largely positive experience. This sat in contrast with the reflections of young people, which highlighted more problematic educational trajectories. This difference has been attributed to the erosion of stability, as older children have often experienced disrupted learning opportunities because of multiple placement moves (Pecora, 2012; The Fostering Network, 2014) and the associated inadequate information transfer between agencies (Zetlin *et al*., 2004). However, longitudinal studies are required to effectively map the journeys of LACYP through the educational system, to understand the complexity of how these changes emerge.

Nonetheless, importantly, the barriers to educational attainment that LACYP emphasised accentuate risk factors beyond the commonly documented negative impact of placement moves. Attention to LACYP's in‐depth accounts of their everyday educational experiences highlighted barriers at the micro level, which can be influenced. The points raised in relation to visibility, being made to feel different and low expectations can be addressed through changes in practice, and these changes can potentially enable more positive educational journeys.

Furthermore, LACYP are not simply passive recipients of their ascribed academic identities. Rather, the paper centralised the agency of LACYP in relation to their active rejection of this attribution and their construction of new identities, which draw on successful subject positions. Nevertheless, the establishment of the successful academic subject was contingent on the support of carers or other significant adults in LACYP's educational trajectories. Accordingly, barriers to educational achievement do not necessarily lay within the individual, and agentic subjects can challenge this marginalised positioning; but LACYP still required some form of support from their carers or personalised forms of tailored provision to secure success in maintaining extended academic journeys.

In response to the unintended harms associated with their identity construction within schools, LACYP offered a number of policy and practice recommendations. Firstly, they critiqued the high visibility of review meetings within school, which risks alienating young people who feel resistant towards the label of ‘looked‐after’, whilst detrimentally impacting their attainment when they are taken out of lessons. This is particularly problematic for LACYP who have already experienced a disrupted education due to difficulties in their birth home and subsequent placement moves (Welbourne & Leeson, 2012; Sebba *et al*., 2015). Participants maintained that meetings related to their ‘looked‐after’ status should be held outside school time, to both limit disruption to their education and minimise the potential of them being continually seen as ‘different’ (Mannay *et al*., 2015).

Secondly, LACYP discussed key adults who had high expectations for them and supported them effectively in their educational journeys. Moreover, for the participants who successfully transitioned into higher education, the social and cultural capital of their carers was crucial. Therefore, it is imperative that both school professionals and carers are knowledgeable about how best to guide and support LACYP throughout their education, particularly at key junctures. In Wales, 34% of foster carers are found to have no educational qualifications, compared to 33% in the general population (Collins & Butler, 2003); this pattern is reflected in UK figures (McDermid *et al*., 2012). However, fewer foster carers are qualified up to degree level compared to the national population of adults of working age (Department for Business Innovation and Skills, 2012).

This suggests that many LACYP may not have a strong foundational base of support, experience or knowledge to draw from within their care placements to enable effective transitions to higher education. Accordingly, training for educators, careers services, social workers and designated teachers with responsibilities for looked‐after children might be considered in relation to countering the propensity for low attainment and career expectations, whilst supporting young people with the academic aspects of completing their education.

## Limitations of the study

As the primary and secondary participants were recruited via foster carers invited by The Fostering Network, the foster carers who brought their children were already voluntarily involved in an organisation that supports and trains foster carers. Consequently, the foster carers who responded were what might be termed ‘engaged foster carers’. This suggests potential bias within the sample, and that engagement with LACYP whose foster carers were not involved might have generated a more differentiated data set. However, the time‐bounded nature of the study and issues of access meant that a wider and more differentiated demographic of LACYP could not be consulted.

Similarly, post‐compulsory education participants were recruited through Voices From Care Cymru and many attend or volunteer with the organisation. Other young people who took part came via local authority groups for young people in care or leaving care. Establishing relationships with organisations or staff within local authorities who support LACYP and care leavers was crucial to the success of getting young people along to events; after young people leave care, it can be hard to make contact if they do not access services, volunteer with organisations or keep in touch with leaving care teams. However, this sampling strategy meant that young people not involved in these formal networks were not represented in the study. Nevertheless, it is significant that this, arguably more engaged, sample presented highly problematic educational experiences. This is important, because it suggests that we have only scratched the surface and that, troublingly, a more differentiated sample could present with increased levels of marginalisation.

## Conclusion

The educational disadvantage of LACYP remains a pressing concern, and the present paper has sought to give a voice to the lived experiences of LACYP in order to understand the complexity of their educational journeys and seek potential remedies. The present study has identified the assignment of the ‘supported’ subject position to young people in care, whereby they are excluded from discourses of success due to a perceived need by schools to minimise academic pressure amidst perceptions of an already chaotic and challenging life. Treatment of LACYP as exceptional, and in need of extra resources, compounds the problem of educational disadvantage by stigmatising these individuals and sometimes diminishing their expectations for themselves. However, it is apparent that many LACYP are educationally aspirational, working to reject the negative labels ascribed to them and identifying their desire to be challenged in the realisation of their potential. This understanding of LACYP's aspirations needs to be embedded into everyday practices and procedures, and effective educational support systems need to be developed in order to support these ambitions.

## Notes

1 The Fostering Network is a fostering charity, providing support and campaigning to improve foster care.

2 Voices From Care Cymru is a charity led and inspired by looked‐after children and care leavers.

3 Spice Innovate provides a system for organisations and individuals to exchange their skills and resources.

4 Two participants placed an addition sign after their number of placements (20+ and 6+), but the base figure was used in the calculation of the mean average.

5 These participants were not included in the calculations. There were seven cases of missing data, one participant recorded the response ‘lots’ and four participants responded ‘unsure’.

6 Children and young people in individual interviews chose their own pseudonyms.

7 Focus group participants in the 16+ category were not assigned individual pseudonyms.
